# 肺段切除术治疗早期非小细胞肺癌的预后研究进展

**DOI:** 10.3779/j.issn.1009-3419.2020.102.21

**Published:** 2020-09-20

**Authors:** 孙银 饶, 联华 叶, 欣 崔, 芩玲 孙, 润 曹, 寿勇 肖, 继琛 杨, 维 王, 光强 赵, 云超 黄

**Affiliations:** 650105 昆明，昆明医科大学第三附属医院，云南省肿瘤医院 胸外一科 Department of Thoracic Surgery, the Third Affiliated Hospital of Kunming Medical University, Kunming 650105, China

**Keywords:** 肺肿瘤, 肺段切除术, 实性结节, 生存预后, 适应证, Lung neoplasms, Segmentectomy, Solid nodules, Survival prognosis, Indication

## Abstract

目前手术是治疗早期非小细胞肺癌（non-small cell lung cancer, NSCLC）最好的方式，随着越来越多的单侧或双侧的多原发肺癌被发现，肺段切除术因对这类肿瘤的治疗有着独特的优势而受到广泛关注。磨玻璃为主的早期NSCLC可以通过肺段切除术获得很好的生存预后，而实性为主的NSCLC具有更高的侵袭性，其治疗手段依然存在争议。随着对肺癌的淋巴结转移途径、影像特征和分子生物学方面的深入研究，这类实性结节如果具备一定的特点也能通过肺段切除术达到根治目的。本文针对影响肺段切除术预后的因素展开综述。

## Abstract (en)

Surgery is currently the most appropriate treatment for early-stage non-small cell lung cancer (NSCLC). Increasing unilateral or bilateral multiple primary lung cancer being found, segmentectomy has attracted wide attention for its unique advantages in the treatment for such tumors. Ground glass opacity dominant early-stage NSCLC is associated with a good prognosis and can be cured by segmentectomy, however, the treatment of solid-dominant NSCLC remains controversial owing to the invasive nature. With the in-depth study on the lymph node metastasis pathway, radiological characteristics and molecular biology of NSCLC, a large part of solid nodules with certain characteristics can also be cured by segmentectomy. This paper reviews the research status and progress about the indication of segmentectomy.

肺癌依然是对中国乃至全世界威胁最大的恶性肿瘤，其发病率和病死率均居第一位，并且呈现逐年上升的态势^[1, 2]^。手术是治疗早期非小细胞肺癌（non-small cell lung cancer, NSCLC）最好的方式，与立体定向放射治疗等非手术治疗方式相比，手术干预有更好的生存预后，同时为病理诊断提供依据^[3, 4]^。在1995年由肺癌研究组（Lung Cancer Study Group, LCSG）开展的一项前瞻性多中心随机对照研究^[5, 6]^显示在T1N0期NSCLC患者中亚肺叶组有更高的局部复发率和更低的总生存期（overall survival, OS）及癌症特异性生存期（cancer-specific survival, CSS），于是认为肺叶切除结合系统淋巴结清扫术是治疗早期肺癌的金标准，亚肺叶切除只能用于妥协性治疗^[7]^。但很多学者对此提出了质疑：①该研究中有30%的患者肿瘤直径 > 2 cm；②亚肺叶切除中包含了约30%的楔形切除病例，因肺段具有相对独立的功能与形态，肺段切除术作为一种解剖性切除，对于引流区域淋巴结的处理上肺段切除与肺楔形切除存在较大区别，不能体现肺段切除的长期效果^[8, 9]^；③许多非外周病灶也进行了亚肺叶切除，这导致较高的局部复发率。

近些年，低剂量计算机断层扫描（low-dose computed tomography, LDCT）在临床的广泛应用，越来越多的肺部小结节以及单侧或双侧的多原发肺结节被发现^[10]^。相比肺叶切除术，肺段切除术的创伤更小，能够保留更多的肺组织，这使得多原发病灶的治疗和将来可能发生的原发肺癌行二次手术成为一种可能，因此肺段切除术获得了越来越多的关注^[11]^。目前，人们反复提出的问题是，对于早期肺癌，肺段切除术能否作为根治术式来替代肺叶切除术，适合肺段切除术的肿瘤有哪些特点。本文针对影响肺段切除术预后的因素展开综述，探讨其最佳的适应症。

## 1 肿瘤大小

CSS是一个较好的评估肿瘤相关预后的指标，Bao等^[12]^在一项*meta*分析中纳入了22篇文献，结果表明对于直径≤2 cm的NSCLC，肺段切除与肺叶切除有着相同的CSS，而对于直径 > 2 cm的NSCLC，肺段切除术后的CSS明显不如肺叶切除术。为了获得临床资料平衡的患者群体，Kodama等^[13]^使用倾向评分匹配法（propensity score matching, PSM）比较了138例接受肺叶切除术或肺段切除术治疗的Ia期（≤2 cm）NSCLC患者，两组的5年OS和无病生存期（relapse free survival, RFS）并无差异。在另外一项大样本研究^[14]^运用PSM处理后同样观察到肺段切除术治疗的临床I期（甚至包括Ib期）NSCLC患者的复发率和生存率并不比肺叶切除术差。另外，Altorki等^[15]^对53例行亚肺叶切除术（包括肺段切除术与楔形切除术）治疗的I期（≤2 cm）NSCLC癌患者进行了长达10年的随访，发现所有复发的病例均发生于楔形切除术后，而肺段切除术后的患者并没有复发的情况。

与上述结果相反的是，一项基于美国国家癌症数据库（National Cancer Database, NCDB）的大样本研究使用PSM分析后观察到直径≤2 cm的NSCLC行肺段切除术后的生存预后明显低于肺叶切除术，接受亚肺叶切除术的患者可能会导致不充分的淋巴结清扫和阳性边缘^[16]^。另外，最近的两项研究^[17, 18]^同样报道了一些不利的结果，对于影像表现为纯实性或实性为主的NSCLC，即使肿瘤直径≤2 cm，肺段切除术后也有较高的局部复发率，生存分析显示RFS和OS均低于肺叶切除术，并且多因素分析表明肺段切除术是RFS的独立危险因素。Nishio等^[17]^将同一肺叶内残余肺段的复发性肿瘤和同侧肺门纵隔淋巴结的复发性肿瘤分别定义为局部性复发（local recurrence）和区域性复发（regional recurrence），他们观察到17例患者因行肺段切除术而导致局部性复发，另有9例患者出现肺段切除术后的区域性复发。另外两项研究^[19, 20]^也观察到多例Ia期患者行肺段切除术后发生局部性复发的情况。甚至有研究^[21, 39]^表明即使在小病灶（≤1 cm）的患者中也会出现同一肺叶内残余肺段（non-primary tumor-bearing segments, NTBS）所包含的淋巴结受累的情况。对于这类患者，肺段切除术无法达到根治目的，存在局部复发的风险，并且影响患者的病理分期，不利于术后辅助治疗的制定。由此可见，单纯根据肿瘤大小来决定手术方式并不合适，其他因素（例如影像学特征的差异、淋巴结的处理方式等）也会对预后产生影响。

## 2 影像学表现

磨玻璃影（ground-glass opacity, GGO）被定义为没有掩盖肺纹理的密度均匀增加的区域^[22]^。通过薄层计算机辅助断层扫描（thin-section computed tomography, TSCT）的GGO成分与肿瘤预后有良好的相关性，而实体成分最大直径与肿瘤最大直径的比值（consolidation/tumor ratio, CTR）是反映肿瘤侵袭特异性最优的指标^[22]^。目前认为GGO为主的早期NSCLC预后较好^[23-26]^，可以通过亚肺叶切除（包括肺段切除和楔形切除）达到根治目的，这些结果已被大量回顾性研究和前瞻性研究所证实^[25-27]^。

相反，对于影像学上表现为实性为主或纯实性的病灶选择何种方式治疗存在较大争议。有多项研究^[19, 28-31]^显示对于实性为主或纯实性的Ia期NSCLC患者，肺段切除术可以达到与肺叶切除术相同的生存预后。另一方面则认为，实性为主或纯实性的肿瘤通常具有更高的侵袭能力，更容易发生血管的侵袭和淋巴结的转移^[32]^。即便是 < 2 cm的肿瘤，如果表现为实性为主或纯实性，生存预后也很差^[33]^。另外两项研究^[17, 18]^也证实了直径≤2 cm并以实性表现为主的NSCLC行肺段切除术治疗后的RFS和OS均低于肺叶切除术。而且最近发表的一篇*meta*分析^[34]^同样表明 < 2 cm且实性为主的NSCLC行肺段切除术后的RFS比肺叶切除术更差。Austin^[35]^和Yoshizawa^[36]^还观察到高侵袭性的组织亚型（实体型或微乳头状型腺癌）与实性结节密切相关，这类肿瘤的预后通常很差。由此看来，影像表现为实性为主或纯实性的病灶选择肺段切除术可能并不合适，当然，这些结果还有待前瞻性研究去进一步证实^[38]^。

## 3 淋巴结转移

Matsumura等^[37]^将病灶所在段的段内淋巴结定义为段区域内叶段淋巴结（adjacent lobar-segmental lymph nodes, aLSNs），在肺段切除术中不易清扫或无法解剖到的叶段淋巴结定义为段区域外叶段淋巴结（isolated lobar-segmental lymph nodes, iLSNs）。肺叶切除术相较于肺段切除术最大的优势是在于能够清除掉这些iLSNs。因此，iLSNs的转移与否是决定能否行肺段切除术的关键。该研究^[37]^报道了307例直径≤2 cm的周围型NSCLC中有9例发生iLSNs转移（转移率约为3%）。在其他相关文献^[21, 39]^中也报道了≤2 cm的肿瘤发生iLSN转移的病例，而且即使是≤1 cm的肿瘤也观察到有iLSNs转移。因此，如果行肺段切除术治疗这类肿瘤，那么复发风险无疑会很大。

同时，Matsumura^[37]^和Wang^[39]^没有观察到孤立的iLSNs转移，也就是说，如果对纵隔淋巴结、肺门淋巴结以及aLSNs进行评估为阴性，那么iLSNs的转移也就不会发生，从而可以进行肺段切除术到达根治目的，而术中评估淋巴结状态为阳性的患者需要立即转为肺叶切除术。而且，对于那些术中淋巴结评估为阴性而术后病理却发现为阳性的患者该如何处理也有文献给出了答案^[30, 40, 41]^，这类患者并不需要再次手术切除残留肺段和淋巴结，因为这类患者通常都是孤立的淋巴结受累，再次手术并不能获益^[42]^。

然而，Yamanaka^[21]^的研究观察到对于Ia期NSCLC即便是术中对纵隔、肺门和叶段淋巴结评估为阴性，仍会有患者因肺段切除术而致iLSNs残留。其原因可能与淋巴结的微转移相关^[43]^，因常规病理检查无法检测到微转移的存在而对手术方式的选择产生影响，微转移是否会影响iLSNs还需要更多的研究去证实。因此，术中对淋巴结的评估十分重要，它能够帮助识别大多数有iLSNs转移的患者，但是仅仅根据术中对淋巴结的评估来决定手术方式可能还不够可靠。

## 4 癌胚抗原（carcinoembr yonic antigen, CEA）水平与最大标准摄取值（maximal standardized uptake value, SUVmax）

多项研究^[44-46]^表明CEA升高的水平与预后关系密切，CEA水平越高，淋巴结发生转移的概率也越大，Inoue^[45]^认为CEA水平较高的患者应避免行亚肺叶切除。Tsutani^[47]^的研究表明对于那些影像表现为实性的临床Ia肺腺癌患者，肿瘤直径 < 0.8 cm或SUVmax < 1.5 g/dL可以作为预测其淋巴结阴性的标准。SUVmax > 5 g/dL则被认为是淋巴结转移的重要预测因子，肺段切除术可能会导致这些患者（SUVmax > 5 g/dL）复发风险增高^[36, 48-50]^。Hattori等^[48]^通过多因素分析也指出，支气管充气征、肿瘤直径和SUVmax是临床预测纯实性结节侵袭性的重要指标，该研究将那些同时满足这3项标准（有支气管充气征表现、直径 < 2 cm、SUVmax < 3.2 g/dL）的纯实性肿瘤定义为“非侵袭性肿瘤”，并观察到“非侵袭性肿瘤”的淋巴结受累概率仅为4%，而且术后的3年OS能达到100%；相比之下，即使是临床分期为Ia期的患者，如果这3项标准都不满足，那么3年的OS只有74.1%。但也有研究^[51]^认为对于高代谢（SUVmax≥3 g/dL）的Ia期NSCLC行肺段切除术与肺叶切除术的RFS和CSS并无差异。其他研究^[52, 53]^还观察到原发肿瘤的SUVmax是腺癌患者的预后因子，而不适用于肺鳞癌患者。因此，SUVmax的预测价值还需在大样本的实验中进一步探讨，同时还需将腺癌和非腺癌病例区分对待。

## 5 切缘距离

尽管先前的研究认为 > 2 cm的距离是安全的^[54, 55]^，但最近的一项研究^[20]^对179例行肺段切除术的Ia期NSCLC患者进行了长期的随访观察，发现其中有5例患者在5年之后才出现切缘的复发，而这些患者的手术切缘均 > 2 cm；同时他们还注意到这其中的4例患者复发的肿瘤类型均为贴壁型腺癌（lepidic adenocarcinoma, LA）。这也说明了LA这类惰性肿瘤生长缓慢且容易在术后病理检查中被遗漏。无独有偶，在Nakao等^[56]^的研究中也观察到，即使切缘 > 2 cm，26例行楔形切除的LA患者中有4例在5年之后发生了切缘复发。这对在肺段切除术中如何保证安全的切缘距离提出了新的警示，同时也提醒对这类惰性肿瘤的随访时间不应少于5年。

## 6 基因分子检测

目前关于基因检测的研究主要集中在肿瘤的治疗方面，随着分子生物学的发展，基因突变状态与预后的相关性研究也随之增多。早期关于基因突变与预后关系的研究大都含有许多晚期或接受过辅助治疗（放化疗及靶向治疗）的病例，不利于观察基因改变对复发率或生存率的直接影响。Izar等^[57]^首次对未进行术后辅助治疗的I期NSCLC患者进行观察，发现*EGFR*突变的患者较野生型的患者有更低的复发率，并且在RFS和OS方面都有着显著的优势，*EGFR*基因突变是一个独立的预测指标。Takamochi等^[58]^同样观察到*EGFR*基因突变是一个有利的预后因素，该研究还表明预后与突变基因亚型（外显子21 L858R突变与外显子19缺失）无关。Nishii等^[59]^同样观察到在病理Ia期腺癌患者中，*EGFR*突变型患者的OS和RFS均显著高于*EGFR*野生型患者，*EGFR*突变被认为是影响RFS的独立因素，血管的侵袭在EGFR野生型患者中更常见。随着下一代高通量测序（next generation sequencing, NGS）的开展，基因检测的效能明显提高，一些罕见突变的检出率也开始上升。Kneuertz等^[60]^应用NGS分析了基因状态对早期肺腺癌的影响，发现*KRAS*、*BRAF*基因突变与更差的RFS和OS相关。另外两项研究^[61, 62]^同样观察到*KRAS*基因突变使复发风险显著增加。相关研究^[63-65]^还发现*EGFR*突变与中低级别的组织亚型（原位癌、微侵润癌、贴壁型以及乳头型为主的腺癌）有密切的相关性，而在实体型的腺癌中较为罕见，*KRAS*突变则常发生在粘液性腺癌中，*BRAF*突变则与乳头状为主的腺癌相关。

尽管基因检测有助于筛选出低侵袭性的肿瘤，但很少能在术前或术中就可以获取这些基因信息，因而对于手术方式的选择，基因检测能提供的帮助非常有限。

## 7 总结

总的来说，大部分纯实性或实性为主的早期NSCLC虽然肿瘤体积小，但恶性潜能高，不推荐行肺段切除术治疗这类肿瘤。但并不是所有表现为实性为主的NSCLC都具有高侵袭性和易转移的特点，如何筛选出这部分病例可能需要结合肿瘤直径、CEA、CTR值以及SUVmax等因素综合考虑。目前对于肺段切除的适应症仍然建议执行NCCN指南的标准^[66]^。现在能收集到的研究均为回顾性研究，混杂因素较多，缺乏说服力。术者操作的熟练程度、肿瘤所在部位的不同都会对预后产生影响^[67, 68]^。因此，肺段切除术能否替代肺叶切除术用于早期NSCLC的治疗，还需要在前瞻性、大样本的随机对照试验中寻找答案^[38, 42, 69]^。
